# Assessing the impact of differences in malaria transmission intensity on clinical and haematological indices in children with malaria

**DOI:** 10.1186/s12936-017-1745-8

**Published:** 2017-03-01

**Authors:** Henrietta E. Mensah-Brown, James Abugri, Kwaku P. Asante, Duah Dwomoh, David Dosoo, Frank Atuguba, David J. Conway, Gordon A. Awandare

**Affiliations:** 1 West African Centre for Cell Biology of Infectious Pathogens and Department of Biochemistry, Cell and Molecular Biology, University of Ghana, Legon, Volta Road, Legon, P. O. Box LG 54, Accra, Ghana; 2 Department of Applied Chemistry and Biochemistry, Faculty of Applied Sciences, University for Development Studies, Navrongo Campus, Navrongo, Ghana; 3 Department of Biostatistics, Room A9, School of Public Health, University of Ghana, Legon, Akilagpa Sawyerr Rd, Legon, P.O. Box LG13, Accra, Ghana; 4 Kintampo Health Research Centre, P. O. Box AH 200, Kintampo, Ghana; 5 Navrongo Health Research Centre, Navrongo, Ghana; 6 London School of Hygiene and Tropical Medicine, Keppel Street, London, WC1E 7HT UK

**Keywords:** Malaria, Severe malarial anaemia, Transmission intensity, Sickle cell trait

## Abstract

**Background:**

Malaria control interventions have led to a decline in transmission intensity in many endemic areas, and resulted in elimination in some areas. This decline, however, will lead to delayed acquisition of protective immunity and thus impact disease manifestation and outcomes. Therefore, the variation in clinical and haematological parameters in children with malaria was assessed across three areas in Ghana with varying transmission intensities.

**Methods:**

A total of 568 children between the ages of 2 and 14 years with confirmed malaria were recruited in hospitals in three areas with varying transmission intensities (Kintampo > Navrongo > Accra) and a comprehensive analysis of parasitological, clinical, haematological and socio-economic parameters was performed.

**Results:**

Areas of lower malaria transmission tended to have lower disease severity in children with malaria, characterized by lower parasitaemias and higher haemoglobin levels. In addition, total white cell counts and percent lymphocytes decreased with decreasing transmission intensity. The heterozygous sickle haemoglobin genotype was protective against disease severity in Kintampo (*P* = 0.016), although this was not significant in Accra and Navrongo. Parasitaemia levels were not a significant predictor of haemoglobin level after controlling for age and gender. However, higher haemoglobin levels in children were associated with certain socioeconomic factors, such as having fathers who had any type of employment (*P* < 0.05) and mothers who were teachers (*P* < 0.05).

**Conclusions:**

The findings demonstrate significant differences in the haematological presentation and severity of malaria among areas with different transmission intensity in Ghana, indicating that these factors need to be considered in planning the management of the disease as the endemicity is expected to decline after control interventions.

## Background

The World Health Organization (WHO) estimates that malaria still causes approximately 212 million cases annually worldwide, with 429,000 deaths, mainly in children below the age of 5 years and pregnant women [1]. The most severe forms of the disease are caused by *Plasmodium falciparum,* which accounts for more than 90% of malaria deaths globally [1]. The commonest life-threatening forms of malaria in children are severe malarial anaemia (SMA) and cerebral malaria (CM). As such, malaria is a leading cause of anaemia in children in endemic areas [2], including Ghana [3], and SMA contributes to over 50% of malaria-related deaths in holoendemic areas [4].

The use of vector control strategies, such as long-lasting insecticide-treated nets (LLITNs) and inside residual spraying (IRS), combined with the use of the efficacious artemisinin combination therapy (ACT), have significantly decreased malaria transmission. This has led to a greater than 50% decline in malaria-related mortality in the last decade, from over a million deaths to under 500,000 annually [1, 5]. Since clinical manifestations of malaria vary with differences in transmission levels [6, 7], it is likely that the decreasing transmission intensities will be accompanied by significant changes in clinical and haematological indicators of disease severity. Studies in north-eastern Tanzania have indicated that decreasing levels of malaria transmission results in an increase in the median age of children with malaria, a reduction in the incidence of malaria and an increase in case fatality [8]. Similar findings in the Gambia and Kenya confirm that average age and risk of fatal disease is highest among children living in low endemic areas, in comparison to areas of high endemicity [7]. Therefore, as many countries deploy malaria elimination strategies, a comprehensive analysis of the impact of decreasing malaria transmission on clinical and haematological indicators of disease is necessary to inform appropriate management of a changing malaria phenotype.

Given the significant effects of common haemoglobinopathies on malaria severity observed in a recent large cohort study [9], a comprehensive study of clinical and haematological parameters must include an investigation of the role of these genetic factors. Haemoglobin S (HbS) and haemoglobin C (HbC) variants are common in malaria-endemic populations in West Africa, with more than a quarter of the population carrying one variant or the other in many areas [9, 10]. Heterozygous carriers of HbS (i.e. sickle cell trait, HbAS) are protected against severe and fatal forms of malaria [11, 12]. This protection is thought to be achieved by preventing the development of high density parasitaemia [13–15], and accelerating the acquisition of natural immunity to malaria in carriers [16]. Studies conducted in Ghana [17] and elsewhere [12, 13, 18] have confirmed the protection conferred by sickle cell trait. However, the protective role of HbC remains has been less frequently demonstrated, although most studies show significant protective effects [19–21], whereas one study did not show significant evidence of protection [22]. In Ghana, studies have shown that HbC protects against malaria, though to a lesser extent compared to HbS [17, 23].

This study took advantage of the significant differences in malaria transmission across ecological zones in Ghana, to analyse the variation in key clinical and haematological parameters in children with malaria exposed to different transmission intensity.

## Methods

### Sample collection

Study participants were recruited in hospitals in three ecologically distinct areas in Ghana, namely: the Ledzokuku Krowor municipal (LEKMA) hospital in Accra; War Memorial Hospital, Navrongo in the Kassena and Nankana districts in Northern Ghana, and Kintampo Municipal Hospital in the Kintampo Municipality in the middle belt of Ghana. Malaria transmission intensity as measured by the entomological inoculation rates [EIR reported as infective bites per person per year (ib/p/y)] are highest in Kintampo (>250 ib/p/y), followed by Navrongo (<200 ib/p/y), and lowest in Accra (<50 ib/p/y) [24–26]. A detailed description of the study sites is presented in a recent report [27].

Children between the ages of 2–14 years who had lived in the community for at least 6 months and presented with symptoms of malaria were screened for malaria with rapid diagnostic tests (RDTs) using a drop of blood from a finger prick. After obtaining informed consent from guardians of RDT-positive children, 0.5 mL of blood was collected into EDTA-coated vacutainer tubes (BD biosciences) for parasitological and haematological analysis.

### Determination of clinical and haematological indices

Haemoglobin levels, erythrocyte indices [red blood cell (RBC) counts, mean corpuscular volume (MCV), mean corpuscular haemoglobin (MCH), mean corpuscular haemoglobin concentration (MCHC), and red cell distribution width (RDW)], leucocyte indices [total white blood cell (WBC) count, percent lymphocytes (LYM%), percent monocytes (MON%), percent granulocytes (GRA%), and platelet indices [platelet count (PLT), mean platelet volume (MPV), and platelet distribution width (PDW)] were analysed using an automated haematology analyser. Thick blood smears were stained with Giemsa and examined for parasites using oil immersion microscopy. Parasite density per µL of blood was determined by counting the number of parasites per 200 WBCs and multiplying by the total WBC count obtained from an automated haematology analyser.

### Determination of haemoglobin types

Haemoglobin types were determined by Hb electrophoresis according to manufacturer’s instructions (Helena Laboratories, Beaumont, TX, USA). Briefly, whole blood was haemolysed using haemolysing reagent containing 0.005 M EDTA with 0.01% potassium cyanide. Patient haemolysates and controls were then loaded on to cellulose acetate plates and electrophoresed at 350 V for 25 min in a Tris–EDTA/boric acid buffer (pH 8.4, ionic strength 0.035). Hb electrophorotypes were identified by comparing to control bands.

### Classification of disease severity

Studies have shown that the WHO definition of severe malarial anaemia of <5.0 g/dL with ≥10,000 parasites/µL is restrictive as it excludes children who do not meet the criteria of >10,000 parasite/µL, but still have potentially fatal malarial anaemia [28]. In order to determine the true association between malarial anaemia and the clinical, haematological and socio-demographic factors examined in this study, a more inclusive criteria for severe malarial anaemia of <5.0 g/dL with any parasitaemia, was used, which has also be described by others [7]. Due to the low sample size of the severe malaria groups, children with severe malarial anaemia were grouped with those with moderate malarial anaemia in order to increase statistical power. Thus children with Hb < 8.0 g/dL with any parasitaemia were grouped as having moderate-to-severe malarial anaemia, since previous studies have identified this group as being at increased risk of malaria associated mortality [29]. Children recruited into this study were, therefore, classified into three categories based on the severity of malarial anaemia. The categories were defined as: (1) Uncomplicated malaria (UM): Children with a malaria-positive smear for *P. falciparum* parasitaemia (of any density) without anaemia (i.e., Hb ≥11.0 g/dL); (2) mild malarial anaemia (MMA): children with a malaria-positive smear for *P. falciparum* parasitaemia (of any density) with 8.0 ≤ Hb < 11 g/dL; (3) Moderate-to-severe malarial anaemia (MSMA): children with a malaria-positive smear for *P. falciparum* parasitaemia (of any density) with Hb <8.0 g/dL. For some analyses, participants were classified according to parasite density, using ≥10,000 parasites/µL as definition for high-density parasitaemia.

### Statistical analysis

Data were analysed using Minitab (version 17). Kolmogorov–Smirnov tests were used to determine normality of the data before application of further tests. One way ANOVA or Kruskal–Wallis tests were applied for across group comparisons for normally and non-normally distributed data respectively. Post-hoc tests of either student t test or Mann–Whitney U tests were applied for pairwise analysis where necessary. Chi square analysis was used to compare categorical variables and determine likelihood ratio for association. Univariate and multivariate regression analyses were carried out to determine the predictors of malaria severity. For all analyses, *P* < 0.05 was considered statistically significant.

## Results

### Comparison of clinical and demographic parameters across transmission areas

To investigate the variation in clinical presentation of malaria as transmission reduces, a total of 568 malaria positive children aged between 2 and 14 years, who were exposed to different intensities of *P. falciparum* transmission (Kintampo > Navrongo > Accra) were enrolled. While there was no difference in the proportion of females recruited at each site (Table 1), children enrolled in Accra were generally older than children recruited in Navrongo (P < 0.001) and Kintampo (P < 0.001). In addition, haemoglobin levels increased with decreasing transmission intensity, and were significantly higher in children in Accra compared to Navrongo and Kintampo (P < 0.001 for both comparisons; Table 1). Parasitaemia levels increased with transmission intensity, with the Kintampo group having the highest parasitaemia (P < 0.001 vs Navrongo and vs Accra). In addition, parasitaemia levels in children in Navrongo were significantly higher than in Accra (P = 0.007; Table 1). These data, therefore, suggest that clinical presentation of malaria shifts towards lower parasitaemias and higher haemoglobin levels as transmission intensity reduces.

**Table 1:** Demographic and clinical characteristics of study participants

	Kintampo	Navrongo	Accra	Total	P value
Number of enrolees	275	144	149	568	
Female, %	44.00	52.78	43.28	46.11	0.174*
Age, years	5.37 (0.20)	5.40 (0.40)	6.246 (0.280)	5.60 (0.16)	<0.001**
Haemoglobin levels, g/dL	9.83 (0.10)	9.95 (0.16)	10.72 (0.14)	10.10 (0.08)	<0.001**
Parasitaemia, parasites/µL	148.435 (12.714)	46.429 (3.977)	37.999 (3.274)	101.210 (10.056)	<0.001**

Relative malaria transmission intensities as measured by entomological inoculation rates in the areas are in the order Kintampo > Navrongo > Accra

Data for age, haemoglobin, and parasitaemia are presented as means (standard error of the mean, SEM)

* *P* value obtained from Chi square test, ** P value obtained from ANOVA

### Comparison of haematological parameters in children with malaria across transmission areas

The associations between transmission intensity and haematological indices was also investigated by comparing erythrocyte, leucocyte, and platelet indices in children with malaria across the study sites. RBC counts were significantly higher in areas of lower transmission, mirroring the pattern observed for haemoglobin levels (P < 0.001 for both Accra vs Navrongo and Accra vs Kintampo; Table 2). However, MCV was lowest in the area of lowest transmission, with levels in children in Accra being significantly lower than those in Navrongo (P < 0.001) and Kintampo (P < 0.001; Table 2). MCHC and RDW did not appear to directly relate to transmission intensity, with both indices showing the highest levels in Navrongo (P < 0.001 for across group comparisons for both indices, Table 2). Total WBC counts, as well as percentage lymphocytes in children with malaria decreased with decreasing transmission intensity across the three sites (P = 0.029 and P < 0.001, respectively, Table 2). Percentage monocytes was significantly lower in children in Navrongo, compared to children in Kintampo (P < 0.001) and Accra (P < 0.001), while percentage granulocytes was lowest in Kintampo, compared to children in Navrongo (P < 0.001) and Accra (P < 0.001). Platelet counts and PDW were both highest in Navrongo (P = 0.008 and P < 0.001 across groups, respectively, Table 2), suggesting no direct relationship with transmission intensity. MPV increased with decreasing malaria transmission levels, with children from Accra showing significantly higher levels relative to Navrongo (P < 0.001) and Kintampo (P < 0.001; Table 2).

**Table 2:** Comparison of haematological indices in children with malaria across three transmission areas

Haematological indices	Study sites			Kruskal–Wallis
	Kintampo (N = 266)	Navrongo (N = 144)	Accra (N = 117)	*P* value
	Median (IQR)	Median (IQR)	Median (IQR)	
Erythrocyte indices				
RBC (10^3^/mm^3^)	4.0 (3.5–4.4)	4.1 (3.6–4.5)	4.4 (4.0–4.8)	<0.001
MCV (µm^3^)	78.0 (74.0–81.0)	75.0 (70.0–78.0)	73.7 (68.5–77.7)	<0.001
MCH (pg.)	25.5 (23.6–26.7)	25.8 (23.9–27.3)	25.1 (23.1–26.9)	0.131
MCHC (g/dL)	32.4 (31.7–33.3)	34.3 (33.5–35.2)	34.1 (33.1–35.0)	<0.001
RDW (%)	15.1 (14.1–16.5)	16.4 (15.4–17.6)	14.7 (13.6–16.2)	<0.001
Leucocyte indices				
WBC (10^3^/mm^3^)	8.5 (6.6–13.0)	8.2 (5.8–10.4)	7.5 (5.5–10.3)	0.016
LYM (%)	30.1 (21.0–41.3)	19.4 (13.2–28.9)	18.5 (13.8–29.7)	<0.001
MON (%)	6.8 (5.3–8.6)	5.5 (3.8–7.5)	7.1 (4.5–11.0)	<0.001
GRA (%)	62.6 (49.5–72.6)	75.2 (64.2–83.1)	72.4 (61.6–79.7)	<0.001
Platelet indices				
PLT (10^3^/mm^3^)	125.5 (87.8–182.0)	141.0 (100.0–210.3)	126.5 (78.3–210.3)	0.048
MPV (µm^3^)	7.3 (6.8–8.3)	7.8 (7.3–8.3)	9.3 (8.8–10.2)	<0.001
PDW (%)	15.1 (13.2–17.3)	14.7 (11.5–16.5)	11.9 (10.8–14.0)	<0.001

Data are presented as medians (first quartile-third quartile)

Relative malaria transmission intensities as measured by entomological inoculation rates in the areas are in the order Kintampo > Navrongo > Accra

### Impact of haemoglobin S and C on clinical and haematological indices

The impact of haemoglobins S and C on the clinical and haematological indicators of disease severity was investigated in the entire cohort of children with malaria. Haemoglobin levels differed between children with HbAS compared to HbAA genotype (P = 0.033). However, there was no significant difference in haemoglobin levels in children with HbC genotypes relative to those with the HbAA genotype (P = 0.975; Table 3). RBC counts were not significantly different across the haemoglobin genotypes, but RDW values were relatively higher in the HbAS group relative to the HbAA and HbAC genotypes (Table 3). On the other hand, MCV and MCH levels were lower in the HbAS and HbAS groups relative to the HbAA group. Platelets counts were significantly higher in HbAS relative to HbAA (P = 0.020), however, there was no difference between the HbAC and HbAA groups (P = 0.567; Table 3). MPV levels were lower in the HbAS and HbAC groups relative to HbAA. The remaining indices were not significantly different across the groups (Table 3).

**Table 3:** Relationship between haemoglobin types and haematological indices in children with malaria

Haematological indices	Haemoglobin type			*P* value
	AA (N = 346)	AS (N = 46)	AC (N = 37)	
	Median (IQR)	Median (IQR)	Median (IQR)	
Erythrocyte indices				
Haemoglobin (g/dL)^a^	10.3 (9.0–11.4)	10.80 (9.4–11.65)	10.30 (8.57–11.55)	0.219^#^
RBC (10^3^/mm^3^)	4.1 (3.7–4.5)	4.3 (3.9–4.6)	4.2 (3.6–4.8)	0.187
MCV (µm^3^)	77.0 (73.0–80.0)	74.5 (71.8–77.9)	73.5 (68.7–79.0)	0.007
MCH (pg.)	25.6 (23.8–26.9)	24.5 (23.7–26.6)	24.7 (22.7–25.7)	0.008
MCHC (g/dL)	33.3 (32.2–34.4)	33.4 (32.2–34.4)	32.8 (32.1–34.3)	0.743
RDW (%)	15.4 (14.3–16.7)	16.2 (15.0–17.4)	15.6 (14.8–17.4)	0.002
Leucocyte indices				
WBC (10^3^/mm^3^)	8.2 (6.2–10.5)	8.9 (6.1–11.0)	9.0 (6.9–13.0)	0.058
LYM (%)	27.3 (17.1–40.5)	26.3 (17.4–41.3)	27.0 (15.9–44.0)	0.373
MON (%)	6.3 (4.8–8.6)	6.4 (5.1–8.5)	6.4 (5.1–8.3)	0.253
GRA (%)	64.5 (49.5–76.4)	63.7 (51.7–76.9)	62.7 (44.6–74.8)	0.226
Platelet indices				
PLT (10^3^/mm^3^)	141 (92.0–211.0)	166.5 (106.0–231.3)	137.0 (79.0–234.0)	0.044
MPV (µm^3^)	7.8 (7.3–8.5)	7.6 (6.9–8.1)	7.5 (7.1–8.5)	0.043
PDW (%)	14.6 (12.4–16.7)	14.4 (11.5–16.3)	14.4 (11.9–18.7)	0.555

Data are presented as medians (first quartile-third quartile)

^#^ *P* value obtained from ANOVA

^a^Sample sizes for haemoglobin levels were AA = 431, AS = 49 and AC = 49

**Fig. 1:**
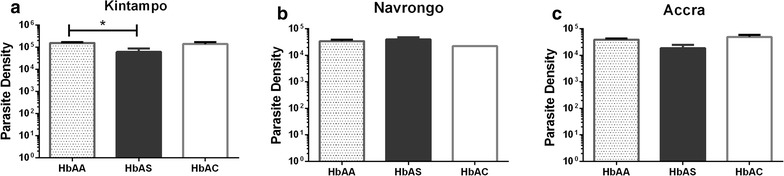
Impact of sickle cell trait on high density parasitaemia infection. Parasite densities in malaria positive children with HbAA, HbAS and HbAC haemoglobin genotypes in **a** Accra **b** Navrongo and **c** Kintampo were compared to determine the impact of sickle cell trait on high density parasitaemia infections. Statistical significance was ascertained by comparing each of the HbAS and HbAC groups with the HbAA group, using Mann–Whitney U test

Additional analysis examined the relationship between haemoglobin genotypes and peripheral parasite densities in children recruited from the three study sites separately. These analyses showed a protective effect of HbAS (Fig. 1), with parasite densities significantly lower in children with HbAS compared to the HbAA group (P = 0.016) in children from Kintampo, although the difference was not significant at the other sites.

### Determinants of malaria severity

**Fig. 2:**
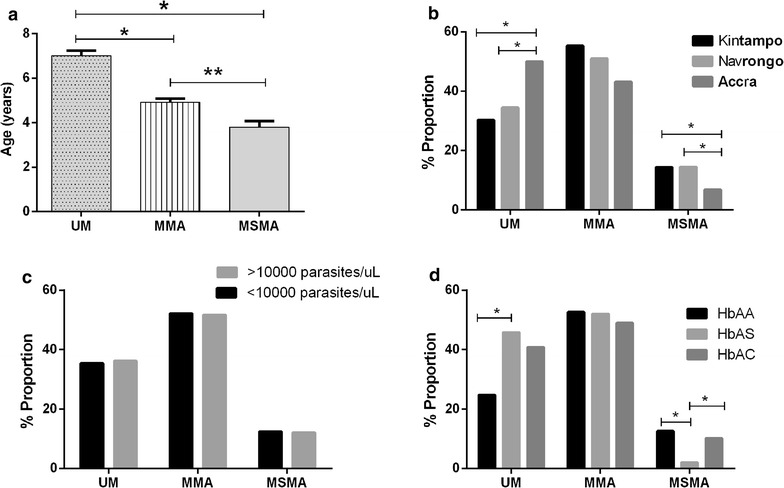
Determinants of malarial anaemia severity in children. Children with malaria were divided into three categories based on haemoglobin levels: UM (Hb ≥ 11 g/dL, n = 194), MMA (8.0 ≤ Hb < 11 g/dL, n = 279) and MSMA (Hb < 8.0 g/dL n = 65). The relationships between severity of malarial anaemia and **a** age **b** study area **c** parasite density and **d** haemoglobin genotype were investigated using Kruskal–Wallis test for age and Chi square analysis for study area, parasite density categories, and haemoglobin genotypes

Since disease severity in this cohort was generally characterized by anaemia, the children with malaria were stratified into three categories based on haemoglobin levels: UM (Hb ≥ 11 g/dL, n = 194), MMA (8.0 ≤ Hb < 11 g/dL, n = 279) and MSMA (Hb < 8.0 g/dL n = 65). The relationship between malaria severity and various demographic, clinical and haematological parameters was then examined by comparing across the malaria groups. Children with UM were significantly older than children with MMA (P < 0.001) and MSMA (P < 0.001; Fig. 2a). Children with MSMA were also significantly younger than children with MMA (P = 0.003; Fig. 2a). Comparison across areas suggests that lower transmission was associated with less severity of malarial anaemia, such that the proportion of patients from Accra who had MSMA was significantly lower than that observed in Navrongo (P = 0.002) and Kintampo (P < 0.001; Fig. 2b), although differences in age distributions of children sampled in the different sites may account for this. Conversely, the proportion of children with UM in the Accra group was significantly higher than the corresponding proportions in Navrongo (P = 0.004) and Kintampo (P < 0.001; Fig. 2b). There were no significant differences in the proportions of children with MMA across the three sites (P = 0.104).

The relationships of anaemia severity with parasite density and haemoglobin types was also investigated. There was no significant relationship between high density parasitaemia (≥10,000 parasites/µL) and anaemia severity. There was however, evidence of the protective effects of HbAS, such that children with this genotype had a significantly lower proportion of MSMA (P = 0.011) and a higher proportion of UM (P < 0.001) compared to the HbAA group (Fig. 2d). There were no significant differences in proportions of disease in the HbAC group compared to HbAA group (P = 0.685, Fig. 2d), indicating that the protective effects were specific for HbAS only.

### Relationship between haematological factors and anaemia severity

To determine indicators of disease severity, haematological indices were compared across the three categories. With the exception of RDW, all red cell indices examined were lower in the malarial anaemia groups compared to UM (Table 4), which was expected since anaemia is primarily a red cell-related disease. In particular, RBC counts and MCV decreased with increasing anaemia severity across the groups (P < 0.001 and P = 0.003; Table 4). In addition, MCH levels were significantly lower in children with MMA (P < 0.001) and MSMA (P < 0.001) compared to the UM group P = 0.002). MCHC levels were significantly lower in children with MMA compared to children with UM (P < 0.001); this was not the case in children with MSMA compared to children in the UM group (P = 0.075). However, RDW increased with increasing anaemia severity across the groups (Table 4), indicating an erythropoietic response. Leucocyte and platelet indices, including, GRA percent, PLT counts and PDW) also decreased with increasing anaemia severity, while LYM and MON percentages increased across the groups (Table 4). Total WBC counts were not significantly different in any of the malarial anaemia groups when compared to children with UM (Table 4).

**Table 4:** Relationship between haematological indices and severity of anaemia in children with malaria

Haematological indices	Severity of malarial anaemia			Kruskal–Wallis
	UM (N = 194)	MMA (N = 279)	MSMA (N = 65)	
	Median (IQR)	Median (IQR)	Median (IQR)	*P* value
Erythrocyte indices				
RBC (10^3^/mm^3^)	4.5 (4.3–4.8)	3.9 (3.5–4.2)	2.5 (2.3–2.8)	<0.001
MCV (µm^3^)	77.6 (73.0–81.0)	75.0 (68.7–79.0)	72.0 (65.3–79.8)	0.003
MCH (pg.)	26.2 (24.8–27.4)	24.8 (22.8–26.6)	24.9 (22.1–26.3)	<0.001
MCHC (g/dL)	33.7 (32.5–34.6)	33.1 (32.1–34.3)	33.3 (31.4–34.9)	0.005
RDW (%)	14.8 (13.8–15.7)	15.8 (14.6–17.1)	17.3 (15.4–19.5)	<0.001
Leucocyte indices				
WBC (10^3^/mm^3^)	8.5 (6.6–11.2)	8.3 (6.1–10.6)	7.6 (4.2–9.8)	0.113
LYM (%)	18.1 (12.0–27.0)	26.7 (18.0–37.2)	39.9 (32.4–48.4)	<0.001
MON (%)	5.7 (3.9–7.7)	6.3 (5.0–8.8)	8.3 (6.6–10.1)	<0.001
GRA (%)	75.6 (65.2–83.6)	66.6 (54.7–76.0)	51.0 (45.1–60.1)	<0.001
Platelet indices				
PLT (10^3^/mm^3^)	168.0 (112.3–229.0)	119.0 (78.0–180.0)	117.0 (69.3–177.8)	<0.001
MPV (µm^3^)	7.9 (7.2–9.0)	7.6 (7.0–8.3)	7.7 (7.2–8.3)	0.005
PDW (%)	14.7 (12.6–17.0)	14.4 (11.6–16.6)	13.5 (10.1–15.5)	0.012

Data are presented as medians (first quartile-third quartile)

### Socio-demographic predictors of anaemia severity and parasitaemia

Multiple regression analyses were performed to determine the socio-demographic predictors of haemoglobin level and parasitaemia, as these are the main clinical indicators of anaemia severity in this cohort. Although several factors were associated with haemoglobin levels in univariate analyses (unadjusted regression coefficients; Table 5), after adjusting for covariates including sickle cell trait and age, it was seen that the father’s educational level as well as the occupations of both parents were significant predictors of haemoglobin levels (adjusted regression coefficients; Table 5). Females were more likely to have lower Hb levels, whilst children whose fathers had middle school education, or better, had higher haemoglobin levels (Table 5). Furthermore, children whose mothers were teachers, and those whose fathers had any kind of employment, were less likely to be anaemic (Table 5). More interestingly, after adjustment for covariates including age, transmission intensity was not a significant predictor of haemoglobin levels.

**Table 5:** Simple and multiple linear regression analysis of factors associated with haemoglobin and log parasitaemia in malaria positive children

	Haemoglobin level in g/dL				Log parasite density			
	β* (95% CI β*)	*P* value	β** (95% CI β**)	*P* value	β* (95% CI β*)	*P* value	β** (95% CI β**)	*P* value
Age	0.21 (0.16, 0.25)	<0.0001	0.19 (0.14, 0.24)	<0.001	−0.07 (−0.09, −0.03)	<0.0001	−0.07 (−0.10, −0.05)	0.002
Sex								
Male	Ref		Ref		Ref		Ref	
Female	−0.29 (−0.61,0.03)	0.079	−0.30 (−0.61, −0.01)	0.055	−0.09 (−0.25, 0.07)	0.267	−0.01 (−0.17, 0.15)	0.896
Ecological zones								
Accra	Ref		Ref		Ref		Ref	
Navrongo	−0.69 (−1.11, −0.34)	<0.001	0.10 (−0.54, 0.73)	0.769	0.29 (0.11, 0.51)	0.003	0.30 (−0.03, 0.63)	0.070
Kintampo	−0.90 (−1.26, −0.52)	<0.001	−0.27 (−0.92, 0.38)	0.418	0.31 (0.11, 0.46)	0.002	0.41(0.08, 0.75)	0.014
Mother’s education								
None	Ref		Ref		Ref	Ref		
Primary	−0.13 (−0.36, 0.62)	0.603	−0.49 (−1.00, 0.03)	0.064	−0.28 (−0.50, −0.02)	<0.050	−0.10 (−0.34, 0.20)	0.447
Middle	0.30 (−0.43, 1.03)	0.425	−0.60 (−1.31, 0.12)	0.104	−0.11 (−0.47, 0.26)	0.563	0.05 (−0.32, 0.43)	0.776
JHS	0.39 (−0.02, 0.79)	0.062	−0.32 (−0.79, 0.15)	0.184	−0.10 (−0.29, 0.10)	0.348	0.03 (−0.20, 0.29)	0.799
Vocation	0.80 (−0.33, 1.94)	0.165	−0.56 (−1.70, 0.59)	0.339	−0.23 (−0.81, 0.35)	0.432	0.16 (−0.43, 0.75)	0.603
Secondary	0.70 (−0.15, 1.24)	0.012	−0.40 (−1.05, 0.25)	0.223	−0.08 (−0.35, 0.19)	0.571	0.01 (−0.32, 0.35)	0.999
Tertiary	1.00 (−0.13, 2.14)	0.083	−0.57 (−1.95, 0.81)	0.416	−0.06 (−0.62, 0.50)	0.830	−0.22 (−0.91, 0.51)	0.551
Father’s education								
None	Ref		Ref		Ref		Ref	
Primary	0.24 (−0.32, 0.79)	0.400	0.27 (−0.27, 0.81)	0.326	0.02 (−0.26, 0.29)	0.991	0.04 (−0.24, 0.32)	0.790
Middle	1.01 (0.51, 1.64)	<0.0001	0.77 (0.18, 1.36)	0.010	0.12 (−0.19, 0.43)	0.666	0.12 (−0.19, 0.43)	0.436
JHS	0.64 (0.17, 1.12)	<0.0001	0.35 (−0.20, 0.91)	0.212	−0.10 (−0.39, 0.19)	0.034	−0.11 (−0.39, 0.17)	0.442
Vocation	0.21 (−1.27, 1.69)	0.780	0.74 (−0.67, 2.16)	0.304	−0.79 (−1.52, 0.06)	0.299	−0.78 (−1.51, −0.05)	0.036
Secondary	1.05 (0.60, 1.51)	<0.0001	0.64 (0.10, 1.18)	0.021	0.03 (−0.26, 0.31)	0.397	0.04 (−0.24, 0.32)	0.770
Tertiary	1.12 (0.56, 1.68)	<0.0001	0.43 (−0.41, 1.27)	0.318	0.17 (−0.26, 0.61)	0.456	0.18 (−0.25, 0.62)	0.406
Mother’s occupation								
Unemployed	Ref		Ref		Ref		Ref	
Farmer	−0.60 (−1.17, −0.03)	0.041	−0.18 (−0.79, 0.43)	0.558	0.26 (−0.02, 0.55)	0.070	0.14 (−0.18, 0.45)	0.390
Teacher	0.82 (0.20, 1.41)	0.114	1.27 (0.23, 2.31)	0.017	0.38 (−0.12, 0.89)	0.138	0.49 (−0.05, 1.03)	0.074
Trader	0.47 (−0.07, 1.0)	0.088	0.41 (−0.12, 0.94)	0.127	0.12 (−0.14, 0,39)	0.368	0.17 (−0.10, 0.45)	0.208
Other	0.14 (−0.47, 0.75)	0.654	0.18 (−0.45, 0.81)	0.567	0.17 (−0.14, 0.48)	0.276	0.18 (−0.11, 0.53)	0.204
Father’s occupation								
Unemployed	Ref				Ref		Ref	
Farmer	0.28 (−0.815, 1.38)	0.613	1.32 (0.08, 2.56)	0.037	0.15 (−0.41, 0.71)	0.602	0.08 (−0.56, 0.72)	0.807
Teacher	1.41 (0.22, 2.60	0.020	1.84 (0.48, 3.20)	0.008	−0.01 (−0.61, 0.63)	0.983	−0.08 (−0.79, 0.62)	0.818
Trader	0.67 (−0.46, 1.80)	0.247	1.33 (0.09, 2.57)	0.036	−0.01 (−0.59, 0.58)	0.990	0.01 (−0.64, 0.65)	0.909
Other	1.00 (−0.11, 2.10)	0.077	1.24 (0.01, 2.46)	0.048	0.02 (−0.55, 0.58)	0.959	0.13 (−0.51, 0.76)	0.456
Professional	0.91 (−0.31, 2.14)	0.143	1.24 (−0.16, 2.65)	0.082	−0.06 (−0.70, 0.58)	0.863	−0.07 (−0.79, 0.66)	0.955
Cement block house								
Yes	Ref		Ref		Ref			
No	−0.40 (−0.72, −0.0)	0.015	−0.07 (−0.44, 0.30)	0.707	−0.11 (−0.05, 0.26)	0.190	0.20 (−0.01, 0.39)	0.045
Thatched house								
Yes	Ref		Ref		Ref			
No	0.64 (0.33, 0.96)	<0.0001	0.17 (−0.24, 0.57)	0.420	−0.03 (−0.19, 0.12)	0.696	0.10(−0.11, 0.31)	0.356
Mosquito control measures								
Bed net use	Ref		Ref		Ref		Ref	
No bed net use	0.69 (0.36, 1.02)	<0.0001	0.30 (−0.09, 0.69)	0.131	−0.40 (−0.56, −0.24)	<0.001	−0.32 (−0.52, −0.12)	0.002
Own farm								
Yes	Ref		Ref		Ref		Ref	
No	0.83 (0.502, 1.16)	<0.0001	0.03 (−0.44, 0.49)	0.917	−0.14 (−0.30, 0.03)	0.113	0.21 (−0.03, 0.44)	0.092
Clinical factors								
Parasite density (parasites/µL)	−1 × 10^−6^ (−2 × 10^−6^, −1 × 10^−7^)	0.003	1 × 10^−7^ (−1.6 × 10^−6^, 1 × 10^−7^)	0.593	N/A	N/A		
Haemoglobin type								
AA	Ref		Ref		Ref		Ref	
AS	0.48 (−0.06, 1.02)	0.084	0.61 (0.053, 1.16)	0.032	−0.157 (−0.43, 0.11)	0.254	−0.20 (−0.48, 0.09)	0.177
AC	−0.01 (−0.55, 0.53)	0.972	0.06 (−0.46, 0.59)	0.813	0.192 (−0.08, 0.46)	0.158	0.24 (−0.03, 0.51)	0.085

N/A is Not Applicable

Ref represent the reference category

β* (95% CI β*) and β** (95% CI β**) represent unadjusted and adjusted regression coefficient and their confidence intervals respectively

A significant predictor of parasite density was ecological zone (sampling site) and the self-reported use of bed nets. As expected, children in Navrongo and Kintampo were more likely to have higher parasitaemia, which is consistent with the reported EIR for these areas.

## Discussion

As a result of concerted intervention strategies, malaria transmission in endemic areas has been declining in the last decade, resulting in a cumulative drop in the incidence of malaria cases worldwide [1]. However, since clinical manifestations of malaria appear to be significantly influenced by transmission intensity [6, 7], the impact of the decreasing transmission on clinical and haematological indices during malaria should be an important consideration in planning for the long-term management of the disease. Using the varying transmission intensity across three well-characterized areas in Ghana as a model, data presented here demonstrate that decreasing transmission leads to a shift in the vulnerable group to slightly older children. This shift was accompanied by a reduced disease severity in children with malaria, characterized by lower parasitaemias, higher haemoglobin levels and higher erythrocyte counts. Previous studies conducted in high transmission areas have shown that anaemia severity was not associated with parasite burden [30, 31]. This study confirms that high density parasitaemia is not necessarily a predictor of anaemia severity, as the proportion of children with and without high density parasitaemia was not different in each of the three categories of malaria. This finding is consistent with previous reports in high transmission settings which have shown a lack of association between parasitaemia and severity of malarial anaemia [30–32]. However, studies in low transmission settings have shown a correlation between parasite density and malaria severity [33, 34], indicating the importance of transmission intensity as an important factor in determining malaria severity. Although parasite density did not significantly correlate with haemoglobin levels in any of the three sites in this study, the relationship between the two was still evident in the data showing that children in the higher transmission areas (Kintampo and Navrongo) had much higher parasitaemias and were more susceptible to moderate-to-severe anaemia compared to those in Accra. This suggest a complex interplay of factors involving transmission intensity, parasite density, and haemoglobin levels.

Malarial anaemia severity was significantly associated with erythropenia, lymphocytosis, monocytosis, agranulosis, reticulocytosis and thrombocytopenia. Thrombocytopaenia is commonly associated with malaria, and is a marker of malaria severity [31, 35]. Reduction in platelet count has been associated with splenomegaly [31], a complication in severe malaria that may lead to death. Consistent with previous reports [12–15, 18], the protective effect of sickle cell trait against malarial anaemia was evident in this cohort. Children with the HbAS genotype had a significantly lower rate of MSMA and higher rate of UM compared to those with normal haemoglobin genotype (HbAA). These patterns were not observed in the HbAC genotypic group, indicating that the mechanisms of protection of the two haemoglobinopathies are likely distinct. In vitro studies have demonstrated that there is inhibition of parasite growth and reduced invasion into HbS erythrocytes [11, 36], and that sickle cell trait accelerates the acquisition of natural immunity [16]. Additional analyses of parasitaemia levels showed that the protective effect of HbAS on parasite density appeared to be specific to only the holoendemic area (Kintampo). This apparent disappearance of the protective effect in lower transmission areas may be a potentially important finding, with significant implications for susceptibility to severe disease in sickle cell carriers in endemic areas as control interventions continue to reduce transmission.

Multivariate analysis revealed that the educational status of the father as well as the economic status of the household are major predictors of the severity of anaemia. Typically in African households, decision-making is the responsibility of the father and hence his level of education and understanding of malaria, as well as the availability of funds would determine whether healthcare would be sought early. In addition, better education may influence nutritional choices, such as intake of vitamins and minerals, which have an impact on haemoglobin levels. Recent reports by Diiro and colleagues in Kenya have shown that households headed by males with a higher level of education were at lower risk of malaria [37]. Children of parents with less than secondary level education are more likely to come from low economic backgrounds, which has been shown to put these children at higher risk of severe malaria [38].

## Conclusion

The results presented in this study demonstrate significant changes in the clinical manifestations as well as the determinants of diseases severity of malaria as transmission intensity decreases. As transmission intensity decreases, the average age of children presenting to hospital with malaria increases while the risk of developing anaemia decreases. Children living in households where the head-of household had Middle school education or better were less likely to develop high density parasitaemia.
